# Determination of soybean routine quality parameters using near‐infrared spectroscopy

**DOI:** 10.1002/fsn3.652

**Published:** 2018-04-17

**Authors:** Zhenying Zhu, Shangbing Chen, Xueyou Wu, Changrui Xing, Jian Yuan

**Affiliations:** ^1^ College of Food Science and Engineering, Collaborative Innovation Center for Modern Grain Circulation and Safety Key Laboratory of Grains and Oils Quality Control and Processing Nanjing University of Finance and Economics Nanjing China; ^2^ School of Food Science and Technology Jiangnan University Wuxi China

**Keywords:** mathematical model, near‐infrared spectroscopy, soybean

## Abstract

Large differences in quality existed between soybean samples. In order to rapidly detect soybean quality between samples from different areas, we have developed near‐infrared spectroscopy (NIRS) models for the moisture, crude fat, and protein content of soybeans, based on 360 soybean samples collected from different areas. Compared with whole kernels, soybean powder with particle sizes of 60 mesh was more suitable for modeling of moisture, crude fat, and protein content. To increase the reproducibility of the prediction model, uniform particle sizes of soybeans were prepared by grinding and sieving soybeans with different sizes and colors. Modeling analysis showed that the internal cross‐validation correlation coefficients (*R*_cv_) for the moisture, crude fat, and protein content of soybeans were .965, .941, and .949, respectively, and the determination coefficients (*R* ^2^) were .966, .958, and .958. NIRS performed well as a rapid method for the determination of routine quality parameters and provided reference data for the analysis of soybean quality using FT‐NIRS.

## 1 INTRODUCTION

China is a high consumption country of soybean which has been regarded as the health food (He & Chen, 2013). Soybeans are one of the main agricultural products of China and several hundred varieties are grown, with huge differences in composition that arise from the rich genetic diversity and regional planting (Lam et al., 2010). Currently, traditional analyse methods are used to analyze soybean quality indices, which are moisture, crude fat, and protein content. These methods produce highly accurate results, but the analytical processes are time‐consuming and laborious and the chemical reagents that are used contribute to environmental pollution (Liu, 1997). Alternative rapid and accurate analytical methods are, therefore, urgently needed (Baianu et al., 2012; Martin, 1992; Zhu et al., 2011).

Near‐infrared spectroscopy (NIRS) is a rapid technique that can be used for the simultaneous detection and analysis of multiple components (Acquah, Via, Billor, Fasina, & Eckhardt, 2016; Baianu et al., 2012; Louw & Theron, 2010; Wehling, Pierce, & Froning, 1988; Williams, Norris, & Sobering, 1985). Different chemical components in samples can be rapidly quantified using NIRS by taking advantage of the vibrational absorption modes of the compounds in the NIR region of the spectrum (Martelovidal & Vazquez, 2014). The frequency doubling and combination bands of various hydrogen‐containing groups in moisture, protein, fat, and carbohydrate all fall within the NIR region, and the characteristic vibrational information of the hydrogen‐containing groups in these organic molecules can be used to determine the chemical composition of mixtures (Givens, De Boever, & Deaville, 1997).

NIRS is nondestructive, fast and needs no complicated sample pretreatment. Because of these advantages, the technique has been evaluated as a method for the analysis of many agricultural products, including beef, eggs, apples, and tomatoes (Mitsumoto, Maeda, Mitsuhashi, & Ozawa, 1991; Peirs, Scheerlinck, De Baerdemaeker, & Nicolai, 2003; Slaughter, Barrett, & Boersig, 1996; Uddin & Okazaki, 2004; Wehling et al., 1988). As to the evaluation of the quality of agricultural products, including rice, wheat, corn, rape, and soybean, this technology is widely used (Agelet et al., 2012; Baianu, You, Guo, Costescu, & Prisecaru, 2011; Bao, Cai, & Corke, 2001; Barton, Shenk, Westerhaus, & Funk, 2000; Dowell et al., 2006; Kovalenko, Rippke, & Hurburgh, 2006; Liu et al., 2008; Peiris, Bockus, & Dowell, 2015; Peiris, Dong, Bockus, & Dowell, 2014; Peiris et al., 2010). For the evaluation of soybean quality, AACC International (formerly the American Association of Cereal Chemists) currently recommends near‐infrared reflectance method for protein, crude fat, and moisture content analysis in soybean based on intact seed (International, 2010b). However, a number of factors, including sophistication of instruments, sample particle size, moisture content, temperature, and color will affect the outcomes of experiments (Fernandezahumada et al., 2006). Sample particle size and uniformity have been shown to be the main factors affecting the accuracy of NIR analysis and well‐controlled particle size and uniformity of samples thus provide the basis for the establishment of a good model (Williams & Thompson, 1978). In our study, we found sharp differences among soybeans in terms of grain size and color, especially for the complex and diverse Chinese soybeans, which means that there is a requirement to investigate soybeans qualities in China for the purpose of Chinese standard updating. We decided, therefore, to crush the whole grains of soybeans to determine appropriate particle sizes for the establishment of a diffuse reflectance Fourier transform NIRS (FT‐NIRS) prediction model. Soybean quality index models were established using uniform particle sizes to avoid the problems of poor reproducibility and accuracy caused by different varieties, different growing regions, and different grain sizes of soybean samples and to provide reference data for the analysis of soybean quality using FT‐NIRS.

## 2 EXPERIMENTAL SECTION

### 2.1 Reagents and apparatus

Concentrated sulfuric acid, sodium hydroxide, boric acid, hydrochloric acid, petroleum ether, bromocresol green, methyl red, anhydrous sodium carbonate, potassium sulfate, copper sulfate, and ethanol were all analytical grade (AR) reagents and were purchased from Sigma‐Aldrich Shanghai Trading Co Ltd (Shanghai, China).

MB3600 FT‐NIR spectrometer was purchased from ABB‐Bomem (Quebec, Canada).

### 2.2 Sample Collection

The 360 samples soybeans (total 50 varieties) were collected from two represented areas. Two hundred and forty samples collected from Northeast China through National Research Center of Soybean Engineering and Technology in October 2013. One hundred and twenty samples collected from the Yangtze River through Zhenjiang Grain and Oil Co., Ltd in September 2014. Samples were dried in oven at 25°C for 24 hr.

### 2.3 Preparation of soybean sample sets and classification of model samples

The preparation of soybean sample has 90 samples. Each soybean sample was 500 g, and each sample was divided into two equal parts. One part was stored for future use, and the other part was divided into five equal parts. The soybean samples were crushed using a high‐speed multifunction mill and screened through mesh sizes of 10, 20, 40, 60, and 80 to provide particles with diameters of 2, 0.9, 0.45, 0.3, and 0.2 mm, respectively. When more than 95% of the particles had passed through the mesh sieve, the individual powders were thoroughly mixed and scanned to determine the best particle size for modeling. The remaining 270 soybean samples were crushed and screened using the best method according comprehensive modeling of the first 90 samples and then divided into a calibration set (*n *=* *216) and an external validation set (*n *=* *54) to establish the best model for determination of moisture, crude fat, and protein content.

### 2.4 Chemical analysis of soybean samples

The moisture content of the soybeans was determined according to AACC Method 44‐15.02 (International, 2010c), the crude fat content was determined according to AACC Method 30‐25.01 (International, 2010a), and the protein content was determined according to AACC Method 46‐11.02 (protein was determined by the combustion method, with a protein correction factor of %N × 6.25) (International, 2010d). Each sample was analyzed three times, and the final results are presented as mean values.

### 2.5 Collection of near‐infrared spectra

To ensure consistency of the samples used for NIR scanning, the sample thickness was maintained at 2 cm. A high efficiency MB3600 FT‐NIR spectrometer, with a scanning spectral range of 3700–15,000/cm and built‐in Horizon MB stoichiometric modeling software, was used to collect the spectra of the soybean samples. The spectrometer was turned on and allowed to warm up for 30 min and the spectra were then collected over the range 4000–12,600/cm, at a resolution of 16/cm with 60 scan number, which containing the absorbance regions of the traits of interest (4000–9000/cm for protein, moisture, and fat). Each sample was scanned three times to eliminate differences caused by objective factors.

### 2.6 Evaluation of the NIR model

The performance of the prediction model was evaluated using an internal cross‐validation method, which incorporates root mean square error of calibration (RMSEC), standard error of cross‐validation (SECV), and correlation coefficient of cross‐validation (*R*_cv_). Smaller values of RMSEC and SECV and higher values of *R*_cv_ indicate better performance of the prediction model (Ferreira, Galão, Pallone, & Poppi, 2014). External validation is the evaluation of the predictive performance of the calibration model in the validation sample set. The predictive performance of the model can be evaluated using the determination coefficient (*R* ^2^) and the statistical probability (*p* value). Higher values of *R* ^2^ and *p* values <0.05 indicate better performance of the prediction model.

## 3 RESULTS AND DISCUSSION

### 3.1 Analysis of soybean components

The quality indices, moisture, crude fat, and protein content, for soybeans samples used in the study, are presented in Table 1. For the 90 selected samples, moisture, crude fat, and protein content were 8.47%–10.67%, 17.71%–25.14%, and 37.37%–43.20%, respectively. For the 216 samples in the calibration set, moisture, crude fat, and protein content were 7.42%–13.71%, 15.78%–25.57%, and 37.37%–43.21%, respectively. For the 54 samples in the external validation set, moisture, crude fat, and protein content were 6.92%–11.24%, 17.75%–25.39%, and 37.04%–43.56%, respectively. The number of samples used for modeling was much higher than 50, which is the minimum sample size proposed for NIRS modeling (Williams et al., 1985). The quality indices, moisture, crude fat, and protein content were widely distributed and were representative of sample composition, providing favorable conditions for the establishment of quality models.

**Table 1:** Descriptive statistics of the soybean chemical parameters

Parameters	Sample number(*n*)	Maximum value(%)	Minimum value(%)	Mean value (%)	Standard (%)
Moisture	90	10.67	8.47	9.37	0.46
	216	13.71	7.42	9.30	1.09
	54	11.24	6.92	9.41	0.90
Crude fat	90	25.14	17.71	19.68	1.41
	216	25.57	15.78	21.91	1.95
	54	25.39	17.75	21.89	2.16
Protein	90	43.20	37.37	39.97	1.46
	216	43.20	37.37	40.60	1.02
	54	43.56	37.04	40.54	1.17

### 3.2 Spectrogram of soybean samples

**Figure 1:**
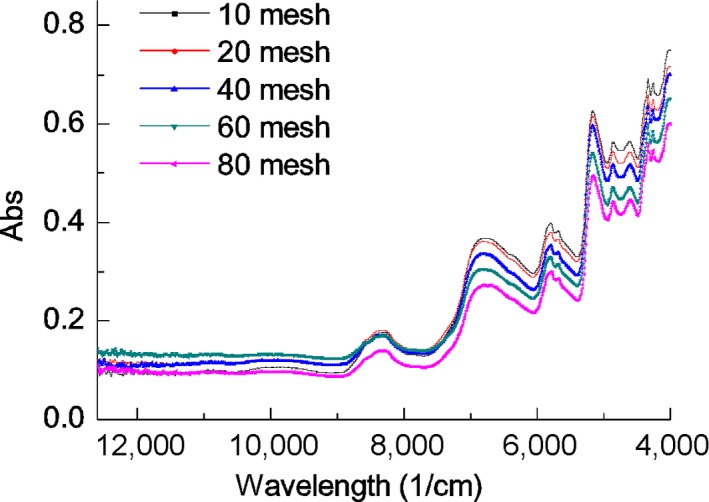
NIR spectra for soybean powder with different mesh

NIRS analysis is based on the characteristic absorption bands from combination vibrational frequencies of NH, CH, OH, and CO in chemical components of samples in the NIR region (Martin, 1992). The position of the absorption bands provides information about the chemical composition of the components, and the strength of the absorption band is proportional to the amount of the hydrogen‐containing group that is present. The NIR spectra of soybean samples can be used as a basis for quantitative analysis of quality indices. The distribution pattern of the sample group under investigation is not accurately reflected if the sample size is too small or too large and useful information may be obscured because irrelevant statistical differences are emphasized. As a result, the performance of the model is greatly reduced. Fewer, but more valuable, samples should thus be chosen to ensure the establishment of a model with the best predictive power. Variations in the intensity of the absorption bands for five samples of the same soybean with different particle sizes at different wavelengths in the spectral region 4000–12,600/cm are shown in Figure 1. The intensity of the absorbance showed a tendency to increase with increasing particle size. Spectral variation was also greater at higher wavelengths, thus affecting the reliability of the NIR prediction model.

### 3.3 Selection of optimal particle size for soybean modeling

Many studies that describe models for evaluation soybean quality have been published. In the research, crushed soybean kernels showed better modeling effect for quality prediction. Pazdernik et al. analyzed the amino acid and fatty acid content of whole grains and crushed samples of soybeans using NIRS technology and obtained cross‐validation *R* ^2^ values of .380–.850 and .060–.830 for the crushed samples and whole grains, respectively, demonstrating the higher accuracy of the tests conducted on crushed samples (Pazdernik, Killam, & Orf, 1997). Haughey et al. analyzed different soya bean meal by NIR, and the correlation coefficient of the model was between 0.990 and 0.890(Haughey, Graham, Cancouët, & Elliott, 2013).

In our paper, we firstly analyzed the moisture, crude fat, and protein content of whole kernels using NIRS technology, and the results indicated that the *R*_cv_ of the moisture content model was .971. However, the *R*_cv_ values of the crude fat and protein models, which were .520 and .495, respectively, showed the predication ability of these two models was low. So modeling analysis of 90 crushed soybean samples with different size was performed using Horizon MB stoichiometric software, combined with partial least squares (PLS) analysis. Samples were pretreated and the data were then processed using appropriate spectral mathematical procedures, including multiple scattering correction, derivation, detrending, normalization, offset correction, and standard normal variate, to determine the optimal particle size for modeling the moisture, protein, and crude fat content of soybeans. Ninety samples of soybean crushed samples were sieved, the particle size were 0, 20, 40, 60, 80 mesh, the establishment of the appropriate model to find the best modeling particle size, the experiment using Horizon MB stoichiometric software modeling results in Table 2.

**Table 2:** The effective of particle size for soybean quality modeling

Parameters	Particle size (mesh)	RMSEC	SECV	*R*_cv_
Moisture	10	0.522	0.273	.954
	20	0.520	0.270	.955
	40	0.503	0.253	.960
	60	0.612	0.374	.914
	80	0.637	0.405	.899
Crude fat	10	0.477	0.228	.895
	20	0.361	0.096	.913
	40	0.281	0.078	.934
	60	0.266	0.071	.939
	80	0.282	0.079	.933
Protein	10	0.554	0.307	.928
	20	0.431	0.186	.930
	40	0.379	0.144	.950
	60	0.390	0.152	.953
	80	0.409	0.167	.948

**Figure 2:**
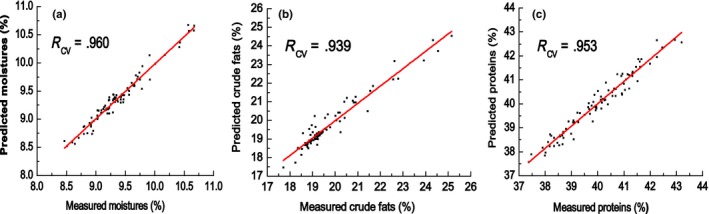
Plot of the predicted values by NIR against the values measured by standard methods for moisture (a), crude fats (b), and protein (c) content of optimal size soybean powder

As shown in Table 2, the *R*_cv_ of the soybean moisture content model increased from .954 to .960 when the particle size of crushed samples was decreased from 10 mesh to 40 mesh. The *R*_cv_ values of the 60 and 80 mesh models, which were .914 and .899, respectively, were lower than that of the 40 mesh model. The *R*_cv_ of the soybean crude fat content model increased from .895 to .939 when the particle size was decreased from 10 mesh to 60 mesh. The *R*_cv_ of the 80 mesh model was .933, which was lower than that of the 60 mesh model. The *R*_cv_ of the soybean protein content model increased from .928 to .953 when the particle size was decreased from 10 mesh to 60 mesh. The *R*_cv_ of the 80 mesh model was .948, which was lower than that of the 60 mesh model. This phenomenon may be induced from the soybean grinding process as long grinding time may lead slight soybean quality changes. Based on an overall consideration of the modeling results of the crushed soybean samples, the standardized multivariate scatter correction method was selected as the best method for determination of the moisture content of soybeans. As shown in Table 2, crushed soybean particles, screened using a 40 mesh sieve, gave the minimum RMSEC and SECV values and the maximum *R*_cv_ value (.503, .253, and .960, respectively). The normalized multivariate scatter correction method was selected as the best method for determination of the crude fat content of soybeans. Crushed soybean particles, screened using a 60 mesh sieve, gave the minimum RMSEC and SECV values and the maximum *R*_cv_ value (.266, .071, and .939, respectively). The deviation‐corrected multivariate scatter correction method was selected as the best method for determination of the protein content of soybeans. Crushed soybean particles, screened using a 60 mesh sieve, gave the minimum RMSEC and SECV values and the maximum *R*_cv_ value (.390, .152, and .953, respectively). The models for moisture, crude fat, and protein content, established under optimal conditions, are shown in Figure 2.

### 3.4 Establishment of NIR calibration model

In this step, the calibration model using 216 soybean samples which were crushed to optimal particles size. The data were then processed using appropriate spectral mathematical procedures mentioned before, including multiple scattering correction (MSC), derivative, detrending, normalization, offset correction, and standard normal variate. The internal cross‐validation method is used to evaluate the predictive performance of the model. The internal cross‐validation verifies the superiority of the detection model by RMSEC, SECV, and cross‐correlation coefficient *R*_cv_. The smaller the RMSEC and SECV, the larger the *R*_cv_, and the better the model predictive performance.

In this study, Horizon MB stoichiometric software modeling and analysis of near‐infrared instrument were used to pretreat the calibration sample. After proper mathematical treatment, it can be seen from Table 3 that for the soybean crushed particles, 40 mesh water RMSEC and SECV were the smallest, *R*_cv_ was the largest, RMSEC was 0.451, SECV was 0.203, and cross‐validation correlation coefficient was 0.965. As can be seen from Table 4, the calibration curve of the normalized normalized multiple scattering of 60‐mesh crude fat for soybean smash particles is the best, RMSEC and SECV are the smallest, *R*_cv_ is the largest, RMSEC is 0.735, the SECV was 0.540, and the cross‐validation correlation coefficient *R*_cv_ was .922. As can be seen from Table 5, the method for correcting the scattering of 60 mesh protein by soybean smash particles is the best using the multiple scattering correction method with the smallest RMSEC and SECV, the largest *R*_cv_ and the standard deviation of correction (RMSEC) of 0.537, the error (SECV) was 0.287, and the cross‐validation correlation coefficient *R*_cv_ was .920.

**Table 3:** Effects of near‐infrared detection model on sieving soybean moisture by different processing methods

	Processing method	RMSEC	SECV	*R*_cv_	*R*
Moisture	MSC	0.464	0.215	.961	.980
	Derivative	0.474	0.224	.958	.979
	Detrending	0.479	0.230	.956	.978
	Normalization	0.466	0.218	.960	.980
	MSC/Derivative	0.458	0.210	.963	.981
	MSC/Detrending	0.462	0.214	.962	.981
	MSC/Normalization	0.466	0.218	.960	.980
	Derivative/Detrending	0.476	0.226	.957	.978
	Derivative/Normalization	0.451	0.203	.965	.983
	Detrending/Normalization	0.454	0.206	.964	.982

**Table 4:** Effects of near‐infrared detection model on sieving soybean crude fat by different processing methods

	Processing method	RMSEC	SECV	*R*_cv_	*R*
Crude fat	MSC	0.735	0.541	.922	.960
	Detrending	0.758	0.574	.912	.955
	Offset correction	0.741	0.549	.920	.959
	Standard normal variate	0.750	0.562	.916	.957
	MSC/Detrending	0.756	0.571	.913	.956
	Offset correction/MSC	0.735	0.541	.922	.960
	Standard normal variate/MSC	0.735	0.540	.922	.960
	Detrending/Offset correction	0.758	0.575	.912	.955
	Detrending/Standard normal variate	0.773	0.597	.905	.952
	Offset correction/Standard normal variate	0.747	0.558	.917	.958

**Table 5:** Effects of near‐infrared detection model on sieving 60 mesh soybean protein by different processing methods

	Processing method	RMSEC	SECV	*R*_cv_	*R*
Protein	MSC	0.537	0.288	.920	.959
	Detrending	0.604	0.365	.871	.933
	Offset correction	0.637	0.406	.840	.917
	Standard normal variate	0.663	0.439	.814	.902
	MSC/Detrending	0.612	0.375	.864	.930
	Offset correction/MSC	0.537	0.287	.920	.959
	Standard normal variate/MSC	0.537	0.288	.920	.959
	Detrending/Offset correction	0.610	0.373	.866	.930
	Detrending/Standard normal variate	0.627	0.393	.851	.922
	Offset correction/Standard normal variate	0.602	0.362	.873	.935

**Figure 3:**
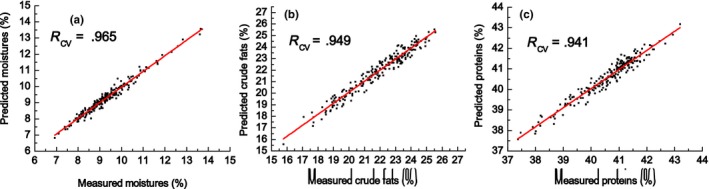
Plot of the predicted values by NIR against the values measured by standard methods for moisture (a), crude fats (b), and protein (c) content based on the results of calibration set after correction

Under the best processing method, the *R*_cv_ values for moisture, crude fat, and protein content were .965, .922, and .920 shown in Table 6. However, some outliers will inevitably occur in the establishment of a prediction model using NIR spectral data, and the presence of these outliers will seriously affect the accuracy of the prediction model. To avoid the elimination of outliers by mistake, the soybean quality value and spectrum of outliers were measured again. If it is still an outlier, it is permanently removed from the calibration set; otherwise, the sample is retained. Results of the corrected NIR calibration model are shown in Table 6 and the data based on the corrected model are shown in Figure 3. The *R*_cv_ values for soybean crude fat and protein content increased to .949 and .941, respectively, after correction. There were no outliers in the NIR model for moisture content in crushed soybeans as the moisture detection based on AACC method or NIR method was simple and effective.

**Table 6:** Chemometrics results of calibration model and correction

Parameters	Processing Method	RMSEC	SECV	*R*_cv_
Moisture	Derivative/Normalization	0.451	0.203	.965
Crude fat	Standard normal variate/MSC	0.735	0.540	.922
Correction of crude fat	Standard normal variate/MSC	0.648	0.420	.949
Protein	Offset correction/MSC	0.537	0.288	.920
Correction of protein	Offset correction/MSC	0.506	0.256	.941

Based on the NIR methods, soybean moisture and protein content models were also established by Ferreira et al. who obtained *R* ^2^ values of .800 and .810 for the moisture content model and protein content model, respectively (Ferreira, Pallone, & Poppi, 2013). D.S. Ferreira used totally 40 soybean samples to use near‐infrared and midinfrared spectroscopy, with diffuse reflectance measurements, associated with multivariate calibration methods based on partial least squares algorithm. The determination coefficient (*R* ^2^) for moisture, ash, protein, and lipid content were 0.72, 0.73, and 0.88, respectively, having an RMSECV (root mean square error of cross‐validation) <2.09% (Ferreira et al., 2014). However, in this study, the calibration model using 216 soybean samples was improved after the samples were crushed to give particles of uniform size with better prediction ability. The cross‐validation *R*_cv_ values for soybean moisture, crude fat, and protein content were .965, .949, and .941, respectively, with very small SECVs (0.203, 0.420, and 0.256, respectively).

### 3.5 External validation of NIR soybean model

SPSS linear regression analysis was performed on selected soybean samples as externally validated data and experimentally determined chemical values. The Anovab variance table was mainly used for the F test of regression linearity. The statistics F means square regression and mean residual sum of square. If the F value is too small, indicating that the explanatory power of the independent variables to the dependent variable is very poor, fitting the regression line is meaningless. If the smaller the probability value sig, the more obvious the linear correlation is.

As can be seen from Table 7, the F of variance of soybean crushed granules moisture, crude fat, and protein is very large, respectively, 1494.903, 1192.713, and 1173.284, Sig values are 0, and the corresponding regression normalized residuals standard PP diagram, it can be seen that the standard PP diagram for each indicator points is basically located in a straight line, indicating that the external verification of several regression lines is meaningful, the establishment of near‐infrared calibration set model is applicable and can be used to accurately determine the unknown quality of the soybean sample content.

**Table 7:** External validation Anova^b^ variance of each soybean quality

Parameters	Model	Sum of squares	*df*	Mean square	*F*	Sig.
Moisture	Regression coefficients	37.389	1	37.389	1494.903	.000^a^
	Residual	1.301	52	0.025		
	Total	38.689	53			
	Regression coefficients	212.231	1	212.231	1192.713	.000^a^
Crude fat	Residual	9.253	52	0.178		
	Total	221.484	53			
Protein	Regression coefficients	58.207	1	58.207	1173.284	.000^a^
	Residual	2.580	52	0.050		
	Total	60.787	53			

a Dependent variables: whole grain moisture, crushed water, crude fat, and protein predictions.

b Predictors: (constant), crushed water, crude fat, and protein measurements.

**Figure 4:**
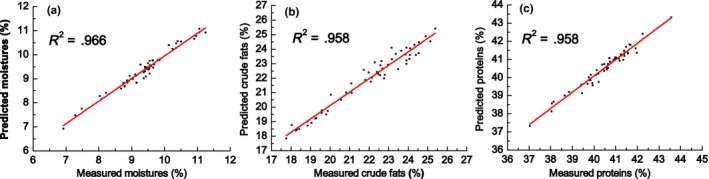
Relation between the real values and the values predicted by the calibration models obtained by NIR for moisture (a), crude fats (b), and protein (c) content based on the results of validation set

External validation results showed that, for the 54 soybean samples as a group, the minimum deviation, maximum deviation, and mean deviation for the moisture content of the crushed grains were 0.004%, 0.349%, and 0.129%, respectively. Corresponding values for crude fat were 0.006%, 0.941%, and 0.329%, and those for protein were 0.012%, 0.607%, and 0.204%. Verification is needed to evaluate established NIR models, and external prediction samples are used to test their applicability. *R* ^2^ values of the soybean quality indices models are close to 1, indicating a high degree of fitting in regression of the measured and predicted values. The Durbin–Watson test is commonly used to detect whether there is a residual. The value of the Durbin–Watson statistic lies between 0 and 4, and a value close to 2 indicates that the variables are independent of each other. We used this test for independent assessment of the residual between the predicted and measured values in the soybean model. Calibration models were imported into the high efficiency MB3600 FT‐NIR spectrometer used in the experiment. Spectral scanning was performed on 54 samples comprising different varieties of soybean collected from Northeast China and the Yangtze River Basin, and predicted values of moisture, crude fat, and protein content were generated. The statistics of the external validation model are shown in Table 8, and correlations between the measured and predicted values are shown in Figure 4.

**Table 8:** Chemometrics results of external validation

	Moisture	Crude Fat	Protein
Minimum deviation (%)	0.004	0.006	0.012
Maximum deviation (%)	0.349	0.941	0.607
Mean deviation (%)	0.129	0.329	0.204
*R*	.983	.979	.979
*R* ^2^	.966	.958	.958
Durbin–watson	1.803	1.838	1.923
*p* value	.000	.000	.000

Many papers have been established good models for fast evaluation of crop quality. Heman et al. established a model to determine the moisture content of rice and obtained an *R* ^2^ value of .920 in external validation of the model (Heman & Hsieh, 2016). Fassio et al. established a model to determine the crude fat content of corn and obtained an *R* ^2^ value of .900 in external validation of the model (Fassio, Restaino, & Cozzolino, 2015). Xiaodong Mao et al. established a model to determine the protein content of wheat and obtained an *R* value 0.975 in external validation of the model (Mao, Sun, Hui, & Xu, 2014). In the present study, *R* ^2^ values of the NIR models for determination of moisture, crude fat, and protein content were .966, .958, and .958, respectively, and *R*_cv_ values were .965, .941, and .949, respectively, demonstrating that the models of soybean moisture, crude fat, and protein content have good predictive value. Values of the Durbin–Watson statistic for the moisture, crude fat, and protein content of crushed soybean grains were very close to 2, indicating that the residual of the model is not self‐correlated and that the regression equation covers the dependent variable changes. Additionally, *p* values for the regression parts were all 0.00, which is lower than the significance level of 0.05, showing that the values predicted by the model are highly significant in the interpretation of real values for soybean samples. After comprehensive analysis using the results of external validation, the predictive performance of the calibration model using the external validation set was found to be credible, indicating that the NIR calibration models for the moisture, crude fat, and protein content of crushed soybean are representative and have good predictive ability.

## 4 CONCLUSION

In this paper, we concluded that achieving a uniform particle size by crushing and sieving provides a good solution to the problem of poor reproducibility of prediction models for soybean quality indices caused by individual differences in samples among varieties. We have studied optimal particle sizes for NIR models of moisture, crude fat, and protein content of soybeans, using FT‐NIRS technology. The external validation results of calibration models using soybean samples from Northeast China and the Yangtze River Basin indicated that models with unified particle sizes showed significant predictive ability for various components in soybean samples of different varieties, from different regions, and with different sizes. Such models showed very high prediction accuracy and reproducibility for soybean moisture, crude fat, and protein content, with external validation *R* ^2^ values of .966, .958, and .958, respectively. Both internal cross‐validation and external validation were performed for these models. The predictive performance of the models, established using the soybean calibration set on samples of the external validation set, was found to be credible, indicating that the NIRS detection models for the determination of the main soybean components are feasible and can be used for rapid determination of the components of soybean.

## CONFLICT OF INTEREST

There was no conflict of interest.

## References

[fsn3652-bib-0001] Acquah, G. , Via, B. , Billor, N. , Fasina, O. , & Eckhardt, L. (2016). Identifying plant part composition of forest logging residue using infrared spectral data and linear discriminant analysis. Sensors, 16, 1375 https://doi.org/10.3390/s16091375 10.3390/s16091375 PMC5038653 27618901

[fsn3652-bib-0002] Agelet, L. E. , Ellis, D. D. , Duvick, S. , Goggi, A. S. , Hurburgh, C. R. , & Gardner, C. A. (2012). Feasibility of near infrared spectroscopy for analyzing corn kernel damage and viability of soybean and corn kernels. Journal of Cereal Science., 55, 160–165. https://doi.org/10.1016/j.jcs.2011.11.002

[fsn3652-bib-0003] Baianu, I. C. , You, T. , Costescu, D. M. , Lozano, P. R. , Prisecaru, V. I. , & Nelson, R. L. (2012). Determination of soybean oil, protein and amino acid residues in soybean seeds by high resolution nuclear magnetic resonance (NMRS) and near infrared (NIRS). Nature Precedings. http://dx.doi.org/10.1038/npre.2012.7053.1.

[fsn3652-bib-0004] Baianu, I. C. , You, T. , Guo, J. , Costescu, D. M. , & Prisecaru, V. I. (2011). Soybean composition database from NIR, NMR and GC‐MS analyses. Nature Precedings. http://dx.doi.org/10.1038/npre.2011.6201.2.

[fsn3652-bib-0005] Bao, J. , Cai, Y. , & Corke, H. (2001). Prediction of rice starch quality parameters by near‐infrared reflectance spectroscopy. Journal of Food Science., 66, 936–939. https://doi.org/10.1111/j.1365-2621.2001.tb08215.x

[fsn3652-bib-0006] Barton, F. , Shenk, J. , Westerhaus, M. , & Funk, D. (2000). The development of near infrared wheat quality models by locally weighted regressions. Journal of Near Infrared Spectroscopy., 8, 201–208. https://doi.org/10.1255/jnirs.280

[fsn3652-bib-0007] Dowell, F. , Maghirang, E. , Graybosch, R. , Baenziger, P. , Baltensperger, D. , & Hansen, L. (2006). An automated near‐infrared system for selecting individual kernels based on specific quality characteristics. Cereal Chemistry., 83, 537–543. https://doi.org/10.1094/CC-83-0537

[fsn3652-bib-0008] Fassio, A. , Restaino, E. , & Cozzolino, D. (2015). Determination of oil content in whole corn (*Zea mays* L.) seeds by means of near infrared reflectance spectroscopy. Computers and Electronics in Agriculture, 110, 171–175. https://doi.org/10.1016/j.compag.2014.11.015

[fsn3652-bib-0009] Fernandezahumada, E. , Garridovaro, A. , Guerreroginel, J. E. , Wubbels, A. , Der Sluis, C. V. , & Der Meer, J. W. M. V. (2006). Understanding factors affecting near infrared analysis of potato constituents. Journal of Near Infrared Spectroscopy., 14, 27–35. https://doi.org/10.1255/jnirs.583

[fsn3652-bib-0010] Ferreira, D. S. , Galão, O. F. , Pallone, J. A. L. , & Poppi, R. J. (2014). Comparison and application of near‐infrared (NIR) and mid‐infrared (MIR) spectroscopy for determination of quality parameters in soybean samples. Food Control, 35, 227–232. https://doi.org/10.1016/j.foodcont.2013.07.010

[fsn3652-bib-0011] Ferreira, D. S. , Pallone, J. A. L. , & Poppi, R. J. (2013). Fourier transform near‐infrared spectroscopy (FT‐NIRS) application to estimate Brazilian soybean [*Glycine max* (L.) Merril] composition. Food Research International., 51, 53–58. https://doi.org/10.1016/j.foodres.2012.09.015

[fsn3652-bib-0012] Givens, D. I. , De Boever, J. L. , & Deaville, E. R. (1997). The principles, practices and some future applications of near infrared spectroscopy for predicting the nutritive value of foods for animals and humans. Nutrition Research Reviews., 10, 83–114. https://doi.org/10.1079/NRR19970006 19094259 10.1079/NRR19970006

[fsn3652-bib-0013] Haughey, S. A. , Graham, S. F. , Cancouët, E. , & Elliott, C. T. (2013). The application of near‐infrared reflectance spectroscopy (NIRS) to detect melamine adulteration of soya bean meal. Food Chemistry., 136, 1557–1561. https://doi.org/10.1016/j.foodchem.2012.01.068 23194562 10.1016/j.foodchem.2012.01.068

[fsn3652-bib-0014] He, F.‐J. , & Chen, J.‐Q. (2013). Consumption of soybean, soy foods, soy isoflavones and breast cancer incidence: Differences between Chinese women and women in Western countries and possible mechanisms. Food Science and Human Wellness, 2, 146–161. https://doi.org/10.1016/j.fshw.2013.08.002

[fsn3652-bib-0015] Heman, A. , & Hsieh, C.‐L. (2016). Measurement of moisture content for rough rice by visible and near‐infrared (NIR) spectroscopy. Engineering in Agriculture, Environment and Food, 9, 280–290. https://doi.org/10.1016/j.eaef.2016.02.002

[fsn3652-bib-0016] International, A. (2010a). Approved methods of analysis, 11th Ed. Method 30‐25.01. Crude fat in wheat, corn, and soy flour, feeds, and mixed feeds. Approved November 3, 1999. St. Paul, MN: AACC International https://doi.org/10.1094/aaccintmethod-30-25.01

[fsn3652-bib-0017] International, A. (2010b). Approved methods of analysis, 11th Ed. Method 39‐21.01. Near‐infrared reflectance method for protein and oil determination in soybeans. Approved November 3, 1999. St. Paul, MN: AACC International https://doi.org/10.1094/aaccintmethod-39-21.01

[fsn3652-bib-0018] International, A. (2010c). Approved methods of analysis, 11th Ed. Method 44‐15.02. Moisture – air‐oven methods. Approved November 3, 1999. St. Paul, MN: AACC International https://doi.org/10.1094/aaccintmethod-44-15.02

[fsn3652-bib-0019] International, A. (2010d). Approved methods of analysis, 11th Ed. Method 46‐11.02. Crude protein – improved Kjeldahl method, copper catalyst modification. Approved November 3, 1999. St. Paul, MN: AACC International https://doi.org/10.1094/aaccintmethod-46-11.02

[fsn3652-bib-0020] Kovalenko, I. V. , Rippke, G. R. , & Hurburgh, C. R. (2006). Determination of amino acid composition of soybeans (Glycine max) by near‐infrared spectroscopy. Journal of Agricultural and Food Chemistry, 54, 3485–3491. https://doi.org/10.1021/jf052570u 19127714 10.1021/jf052570u

[fsn3652-bib-0021] Lam, H. , Xu, X. , Liu, X. , Chen, W. , Yang, G. , Wong, F. , … Wang, B. (2010). Resequencing of 31 wild and cultivated soybean genomes identifies patterns of genetic diversity and selection. Nature Genetics., 42, 1053–1059. https://doi.org/10.1038/ng.715 21076406 10.1038/ng.715

[fsn3652-bib-0022] Liu, K. (1997). Chemistry and nutritional value of soybean components In Liu K. (Ed.), Soybeans: Chemistry, technology, and utilization (pp. 25–113). Boston, MA: Springer US https://doi.org/10.1007/978-1-4615-1763-4

[fsn3652-bib-0023] Liu, F. , Zhang, F. , Jin, Z. , He, Y. , Fang, H. , Ye, Q. , & Zhou, W. (2008). Determination of acetolactate synthase activity and protein content of oilseed rape (*Brassica napus* L.) leaves using visible/near‐infrared spectroscopy. Analytica Chimica Acta., 629, 56–65. https://doi.org/10.1016/j.aca.2008.09.027 18940321 10.1016/j.aca.2008.09.027

[fsn3652-bib-0024] Louw, E. D. , & Theron, K. I. (2010). Robust prediction models for quality parameters in Japanese plums (*Prunus salicina* L.) using NIR spectroscopy. Postharvest Biology and Technology., 58, 176–184. https://doi.org/10.1016/j.postharvbio.2010.07.001

[fsn3652-bib-0025] Mao, X. , Sun, L. , Hui, G. , & Xu, L. (2014). Modeling research on wheat protein content measurement using near‐infrared reflectance spectroscopy and optimized radial basis function neural network. Journal of Food and Drug Analysis, 22, 230–235. https://doi.org/10.1016/j.jfda.2014.01.023

[fsn3652-bib-0026] Martelovidal, M. J. , & Vazquez, M. (2014). Evaluation of ultraviolet, visible, and near infrared spectroscopy for the analysis of wine compounds. Czech Journal of Food Sciences, 32, 37–47.

[fsn3652-bib-0027] Martin, K. (1992). Recent advances in near‐infrared reflectance spectroscopy. Applied Spectroscopy Reviews., 27, 325–383. https://doi.org/10.1080/05704929208018109

[fsn3652-bib-0028] Mitsumoto, M. , Maeda, S. , Mitsuhashi, T. , & Ozawa, S. (1991). Near‐infrared spectroscopy determination of physical and chemical characteristics in beef cuts. Journal of Food Science., 56, 1493–1496. https://doi.org/10.1111/j.1365-2621.1991.tb08623.x

[fsn3652-bib-0029] Pazdernik, D. L. , Killam, A. S. , & Orf, J. H. (1997). Analysis of amino and fatty acid composition in soybean seed, using near infrared reflectance spectroscopy. Agronomy Journal., 89, 679–685. https://doi.org/10.2134/agronj1997.00021962008900040022x

[fsn3652-bib-0030] Peiris, K. H. S. , Bockus, W. W. , & Dowell, F. E. (2015). Near‐infrared spectroscopic evaluation of single‐kernel deoxynivalenol accumulation and fusarium head blight resistance components in wheat. Cereal Chemistry, 91, 25–31. https://doi.org/10.1094/CCHEM-03-15-0057-R

[fsn3652-bib-0031] Peiris, K. H. S. , Dong, Y. , Bockus, W. W. , & Dowell, F. E. (2014). Single‐kernel near‐infrared analysis for evaluating wheat samples for fusarium head blight resistance. Cereal Chemistry., 91, 35–40. https://doi.org/10.1094/CCHEM-11-12-0157-R

[fsn3652-bib-0032] Peiris, K. H. S. , Pumphrey, M. O. , Dong, Y. , Maghirang, E. B. , Berzonsky, W. , & Dowell, F. E. (2010). Near‐infrared spectroscopic method for identification of fusarium head blight damage and prediction of deoxynivalenol in single wheat kernels. Cereal Chemistry., 87, 511–517. https://doi.org/10.1094/CCHEM-01-10-0006

[fsn3652-bib-0033] Peirs, A. , Scheerlinck, N. , De Baerdemaeker, J. , & Nicolai, B. (2003). Starch index determination of apple fruit by means of a hyperspectral near infrared reflectance imaging system. Journal of Near Infrared Spectroscopy., 11, 379–389. https://doi.org/10.1255/jnirs.389

[fsn3652-bib-0034] Slaughter, D. C. , Barrett, D. M. , & Boersig, M. (1996). Nondestructive determination of soluble solids in tomatoes using near infrared spectroscopy. Journal of Food Science., 61, 695–697. https://doi.org/10.1111/j.1365-2621.1996.tb12183.x

[fsn3652-bib-0035] Uddin, M. , & Okazaki, E. (2004). Classification of fresh and frozen‐thawed fish by near‐infrared spectroscopy. Journal of Food Science., 69, 665–668.

[fsn3652-bib-0036] Wehling, R. L. , Pierce, M. M. , & Froning, G. W. (1988). Determination of moisture, fat and protein in spray‐dried whole egg by near infrared reflectance spectroscopy. Journal of Food Science., 53, 1355–1359. https://doi.org/10.1111/j.1365-2621.1988.tb09276.x

[fsn3652-bib-0037] Williams, P. C. , Norris, K. H. , & Sobering, D. C. (1985). Determination of protein and moisture in wheat and barley by near‐infrared transmission. Journal of Agricultural and Food Chemistry, 33, 239–244. https://doi.org/10.1021/jf00062a021

[fsn3652-bib-0038] Williams, P. C. , & Thompson, B. N. (1978). Influence of whole meal granularity on analysis of HRS wheat for protein and moisture by near infrared reflectance spectroscopy. Cereal Chemistry, 55, 1014–1037.

[fsn3652-bib-0039] Zhu, D. , Wang, K. , Zhang, D. , Huang, W. , Yang, G. , Ma, Z. , & Wang, C. (2011). Quality assessment of crop seeds by near‐infrared hyperspectral imaging. Sensor Letters, 9, 1144–1150. https://doi.org/10.1166/sl.2011.1377

